# Race, Gender, and the U.S. Presidency: A Comparison of Implicit and Explicit Biases in the Electorate

**DOI:** 10.3390/bs12010017

**Published:** 2022-01-17

**Authors:** Gemma Anne Calvert, Geoffrey Evans, Abhishek Pathak

**Affiliations:** 1 Nanyang Business School, Nanyang Technological University, 50 Nanyang Avenue, Singapore 639798, Singapore; 2 Nuffield College, University of Oxford, New Road, Oxford OX1 1NF, UK; geoffrey.evans@nuffield.ox.ac.uk; 3 School of Business, University of Dundee, Dundee DD1 4HN, UK; a.z.pathak@dundee.ac.uk

**Keywords:** implicit bias, evaluative priming, sexism, racism, political identity, voting

## Abstract

Recent U.S. elections have witnessed the Democrats nominating both black and female presidential candidates, as well as a black and female vice president. The increasing diversity of the U.S. political elite heightens the importance of understanding the psychological factors influencing voter support for, or opposition to, candidates of different races and genders. In this study, we investigated the relative strength of the implicit biases for and against hypothetical presidential candidates that varied by gender and race, using an evaluative priming paradigm on a broadly representative sample of U.S. citizens (n = 1076). Our main research question is: Do measures of implicit racial and gender biases predict political attitudes and voting better than measures of explicit prejudice? We find that measures of implicit bias are less strongly associated with political attitudes and voting than are explicit measures of sexist attitudes and modern racism. Moreover, once demographic characteristics and explicit prejudice are controlled statistically, measures of implicit bias provide little incremental predictive validity. Overall, explicit prejudice has a far stronger association with political preferences than does implicit bias.

## 1. Introduction and Theoretical Background

Social psychologists have been using measures of implicit bias since the mid-1980s [1]. As Gawronski and Houwer [2] note, these measures “aim to capture psychological attributes (e.g., attitudes, stereotypes, self-esteem) without requiring participants to report a subjective assessment of these attributes”. An important theoretical motivation for their use comes from dual process theory, which posits that human cognition is governed by two relatively distinct processes: an automatic (unconscious) one, and an effortful (conscious) one—what Kahneman [3] calls “system 1” and “system 2”, respectively (see [4]). In recent years, the failure of traditional polls (which rely on conscious or explicit voter-stated intentions) to clearly predict the outcome of elections (e.g., Donald Trump to the Presidency of the United States, and the U.K. Brexit referendum, both in 2016) has prompted renewed interest in nonstandard methods of predicting the outcomes of elections and referenda.

Consequently, there is a growing body of literature on the extent to which measures of implicit bias can predict political attitudes and voting [5,6,7]. On the basis of this literature, a number of scholars have argued that utilizing measures of implicit attitudes can help political scientists to better understand the causes of individual political preferences, especially in socially sensitive contexts [6,7,8]. These scholars point to evidence that measures of implicit bias (e.g., for or against the Democratic Party, or for or against particular Republican candidates) are often statistically significant predictors of political attitudes and voting, even in studies that gauge the political preferences of individuals days or weeks after assessing their implicit biases. Other studies have looked specifically at whether measures of implicit racial bias can predict individual political preferences (see [9] for a brief review). Indeed, the higher observed levels of racism among individuals who identify as politically conservative, combined with the tendency of individuals to give socially desirable responses, should lead one to expect that the measures of implicit racial bias would predict support for conservative parties and candidates, potentially over and above the effects of the demographic characteristics and explicit prejudice [10,11,12].

However, the empirical evidence on the importance of implicit biases to date appears to be somewhat mixed. Finn and Glaser [13] found that an implicit preference for whites over blacks had a significant negative association with voting for Barack Obama, which was rendered nonsignificant when the measures of the emotional responses to candidates were included in the model. Another study by Payne et al. [14] found that implicit prejudice against blacks was a robust predictor of voting for a candidate other than Obama, but was not a significant predictor of voting for John McCain once explicit prejudice had been controlled. Similarly, Pasek et al. [15] found that implicit racism was robustly associated with voting for a nonmajor party candidate, but that its association with voting for John McCain was not robust to controlling for explicit prejudice. More generally, Friese et al. [9] argue that, although measures of implicit bias do significantly predict individual political preferences, including them in multivariate models typically leads to only small improvements in the overall accuracy of prediction (e.g., percentage of correctly classified cases).

One possible explanation for these mixed findings relates to criticism of the specific implicit paradigm (the implicit association test (IAT)), which has been the mainstay of examining implicit biases in the political domain. Since its inception in 1998, the IAT has spawned a vast empirical literature. Among other things, studies have reported that individuals exhibit implicit bias toward those who share their demographic characteristics [16], that implicit bias is associated with measures of explicit attitudes in a number of different domains [17], and that implicit bias predicts political identity and behavior independently of explicit prejudice [10]. However, in the last few years, the IAT has been subjected to a number of criticisms, several of which extend to the concept of implicit bias itself [16,18,19,20]. The predictive validity of the IAT has also been called into question. A meta-analysis by Oswald et al. [21] concluded that, “the IAT provides little insight into who will discriminate against whom and provides no more insight than explicit measures of bias” (see also [22,23,24]). Additionally, it has been argued that implicit bias, as measured using the IAT, may simply reflect familiarity with certain cultural stereotypes, rather than an actual endorsement of them [16]. For example, someone who is familiar with a stereotype that blacks are more likely to be involved in violent crime than whites may be deemed to harbor a subconscious animus against blacks, even though she avowedly rejects that stereotype. Such mixed outcomes prompt further investigation into the utility of implicit measures, including those other than the IAT, in predicting subsequent behavior in the fields of political and social psychology.

One such method is the evaluative priming paradigm that we employ here (see [1,25]). Both the IAT and the evaluative priming paradigm measure implicit bias by calculating the differences in the response latencies for certain combinations of prime stimuli (e.g., white or black faces) and target stimuli, often with a hedonic tone (e.g., positive or negative adjectives). However, the evaluative priming paradigm offers several advantages over the IAT. These include: (i) A relatively shorter task duration (often five times shorter than the IAT), thus minimizing respondent fatigue; (ii) The effect is based purely on response latencies rather than on errors made during the task (response confusion); and (iii) Unlike the IAT, for which there is no clear theoretical explanation as to how or why it works, evaluative priming has a clear theoretical underpinning, namely, associative network theory [26], which includes political decision making [27]. Specifically, unlike the IAT, evaluative priming works because it is based on assumptions that are highly compatible with what is known about how the brain processes information [28]. Neural network models of the brain are based on mental associations—the stronger the association between two concepts (e.g., specific candidates and desirable vs. undesirable traits), the quicker one concept will mentally trigger the other. This, and recent criticisms of the IAT, led us to adopt an evaluative priming paradigm to measure implicit attitudes. This approach has not previously been conducted on gender/race presidential candidate assessments.

## 2. Research Questions

In this study, we sought to extend previous research by investigating implicit bias for and against hypothetical presidential candidates that varied by both race and gender, by using an evaluative priming paradigm and approach.

The 2008 U.S. presidential election witnessed the first black candidate, and the 2016 election witnessed the first female candidate nominated by the major parties. And although the 2020 presidential contest was fought between two elderly white males, it also saw the election of a vice president who is both female and of black and south Asian heritage. Racial and gender representation appears to be broadening at the highest levels of U.S. politics, but there are still biases in the electorate that can mitigate against a level playing field for black and female candidates.

We also extend the range of political orientations included in our analysis beyond the presidential vote to include the respondents’ party affiliations and ideological positions. Our main research question is: Do measures of implicit bias predict political attitudes and voting better than measures of explicit prejudice? Specifically, we sought to test whether implicit bias against black candidates and female candidates would predict conservative ideological positions, as well as Republican identities and voting, better than measures of explicit sexism and racism. For this, we use a fully crossed design varying both the sex and race of potential political candidates simultaneously in order to examine whether implicit attitudes are more predictive of a range of political choices and identities than are explicit prejudices, for both race and gender.

## 3. Method

A total of 1077 subjects were recruited in the United States via the market research firms, Instantly and Survey Sampling International, between October 2014 and July 2015. The firms were briefed to recruit respondents from each of the following preregistered demographics until a sufficient sample of each category was achieved: male/female; black/Caucasian; and self-reported Republican/Democrat voters. Emails were then sent out to their subject pools, and individuals who were eligible (on the basis of their preregistered demographics with SSI) and who responded were invited to participate. Each subject was told what they would be expected to do, was informed that they would be paid a small cash sum for participating (approximately USD 18), and was asked to give informed consent. A copy of the message shown to the subjects during the recruitment process is provided in Appendix A.

Before the implicit bias experiment, subjects were asked to fill out a detailed questionnaire on demographics, political affiliations, past voting behavior, and political knowledge. After the implicit bias experiment, they were asked to complete a battery of items pertaining to sexist attitudes, as well as a battery of items pertaining to racist attitudes. The descriptive statistics for our sample of subjects are displayed in Table 1. As women, black respondents, and those with higher levels of education are overrepresented in our sample, we ran all of our models with and without controls for demographic characteristics in order to test whether any results were driven by nonrepresentativeness.

**Figure 1:**
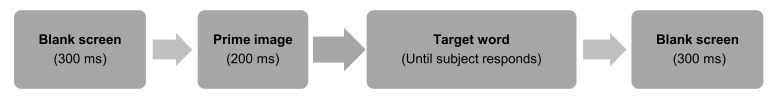
Sequence of events within each trial.

We employed a 2 × 2 × 2 repeated measures design. Each subject participated in 4 blocks of 88 trials, for a total of 352 trials per subject. The sequence of events within each trial is depicted in Figure 1.

Subjects initially saw a blank screen for 300 milliseconds (ms). Next, a prime image was presented for 200 ms. Each prime image depicted a hypothetical presidential candidate who was either a white male, a white female, a black male, or a black female. Next, a target word was presented until the subject responded. Each target word was either a desirable trait (e.g., “honest”) or an undesirable trait (e.g., “dull”). Subjects were instructed to press the “E” key when a desirable trait appeared on the screen, and to press the “I” key when an undesirable trait appeared. An orange rectangle flashed around the target words for 600 ms, and the participants were told to respond before the rectangle disappeared. If a subject took longer than 700 ms to respond on three consecutive trials, a “Too slow” message flashed in red at the bottom of the screen for 500 ms, which reminded the participants to respond faster. After responding, the subjects saw a blank screen for another 300 ms.

In each trial, the length of time taken for the subject to respond (i.e., the response latency) was recorded. This allowed the mean response latency for each combination of treatments to be computed for each subject. The eight combinations of treatments were: male, white, desirable trait; female, white desirable trait; male, black, desirable trait, etc. Response latencies shorter than 200 ms or longer than 900 ms were excluded prior to averaging (e.g., [29]). For respondents whose response latencies were all greater than 200 ms and less than 900 ms, each mean was computed across 44 trials (352 trials divided by 8 combinations of treatments).

Four measures of implicit bias were then constructed (two for gender and two for race): the difference in the time taken to associate males versus females with desirable traits and undesirable traits, respectively; and the difference in the time taken to associate blacks versus whites with desirable traits and undesirable traits, respectively. Let M denote male, *F* denote female, *B* denote black, *W* denote white, *D* denoted desirable, and *U* denote undesirable. Then, *MWD*_i_ denotes Subject i’s mean response latency for trials involving a white male prime image and a desirable target word; *FWD_i_* denotes Subject i’s mean response latency for trials involving a white female prime image and a desirable target word, and so on. The difference in the time taken to associate males versus females with desirable traits for Subject i was therefore computed using the formula:

$$implicit\_bias\_females\_desirable\_traits_{i}=\left[\frac{FWD_{i}+FBD_{i}}{2}\right]-\left[\frac{MWD_{i}+MBD_{i}}{2}\right]$$

The difference in the time taken to associate males versus females with undesirable traits for Subject i was computed using the formula:

$$implicit\_bias\_females\_undesirable\_traits_{i}=\left[\frac{MWU_{i}+MBU_{i}}{2}\right]-\left[\frac{FWU_{i}+FBU_{i}}{2}\right]$$

Assuming that a value of zero represents neutrality, positive values on each measure represent implicit bias against females, while negative values represent implicit bias towards females. The two measures of implicit bias against blacks were constructed in exactly the same way. The raw units of our measures are milliseconds, but we transformed the measures into z-scores for our regression analyses. We obtained the following demographic information on each subject from the pre-experimental questionnaire: gender, race, age group, highest level of education, and income group. In addition, we obtained the subject’s political identity on a 7-point scale, from “strongly liberal” to “strongly conservative”, as well as their party identity (Republican or Democrat) and the candidate for whom they voted in the 2012 presidential election (Mitt Romney or Barack Obama). A neosexism scale was constructed from the postexperimental battery of items pertaining to sexist attitudes [30], and a modern racism scale was constructed from the postexperimental battery of items pertaining to racist attitudes [31]. These two variables constitute measures of explicit sexism and explicit racism, respectively. The scale items are shown in Appendix B. Both of these scales have been validated and have been used extensively to examine social and political attitudes and behaviors. The scale of Tougas et al. [30] has shown impressive predictive validity [32] and has become a standard instrument for measuring sexist attitudes [33]. Likewise, the modern racism scale has become the most commonly used and validated instrument for examining prejudice against blacks in the United States [34,35,36].

## 4. Results

Surprisingly, neither the two measures of implicit bias against women, nor the two measures of implicit bias against blacks, were correlated with one another (r = 0.01, *p* > 0.1). For example, subjects who were quicker to associate women with undesirable traits were no slower, on average, to associate women with desirable traits. Likewise, those who were quicker to associate blacks with undesirable traits were no slower, on average, to associate blacks with desirable traits. This suggests that implicit responses towards negative and positive traits are not necessarily oppositional. However, consistent with previous research, female respondents exhibited slightly less explicit bias against women than male respondents, while black respondents exhibited substantially less explicit bias against blacks than white respondents (see Table 2 and Table 3).

Compared to their white counterparts, black respondents were 0.33sd slower to associate blacks with undesirable traits and were 0.26sd quicker to associate blacks with desirable traits (*p* < 0.001 in both cases). Correlations between measures of implicit bias and measures of explicit prejudice were small in magnitude. Implicit bias against women for undesirable traits was uncorrelated with explicit sexism (r = −0.01, *p* > 0.1), while implicit bias against women for desirable traits was correlated with explicit sexism (r = 0.10, *p* = 0.001). Implicit bias against blacks for undesirable traits was correlated with explicit racism (r = 0.15, *p* < 0.001), while implicit bias against blacks for desirable traits was correlated with explicit racism (r = 0.09, *p* = 0.002).

We now turn to our main results. Table 1 displays the estimates from the OLS models comparing the effects of implicit bias and explicit prejudice on conservative identity: Panel A compares implicit bias against women to explicit sexism; Panel B compares implicit bias against blacks to explicit racism. Explicit sexism and explicit racism have moderate and highly significant effects in all of the models. Neither measure of implicit bias against women enters significantly. Implicit bias against blacks for desirable traits enters significantly but provides only 1 percentage point of additional explanatory power over and above the effects of explicit racism and the demographic characteristics. Table 2 displays the estimates from the logit models comparing the effects of implicit bias and explicit prejudice on Republican identity. Explicit sexism and explicit racism have moderate and highly significant effects in all of the models. Neither measure of implicit bias against women enters significantly. Implicit bias against blacks for desirable traits enters significantly but does not improve the percentage of correctly classified cases relative to the model that just includes explicit racism and demographic characteristics.

Finally, Table 4 displays the estimates from the logit models comparing the effects of implicit bias and explicit prejudice on voting for Romney rather than Obama. Once again, explicit racism and sexism have moderate and highly significant effects in all of the models. Implicit bias against women for desirable traits enters significantly but provides less than 1 percentage point of additional explanatory power. Likewise, implicit bias against blacks for desirable traits enters significantly, but provides no additional explanatory power over and above the effects of explicit racism and the demographic characteristics.

## 5. Discussion

This study investigated implicit bias, for and against hypothetical political candidates that varied by race and gender, using an Internet-based evaluative priming paradigm [37]. We found that the measures of implicit bias were far less strongly associated with political attitudes and voting than were the measures of explicit prejudice. Indeed, our measures of implicit bias offered little incremental predictive validity once demographic characteristics and explicit prejudice had been statistically controlled. Overall, our findings build upon the conclusions of Friese et al. [9] by presenting evidence, for both race and gender, and with respect to three different measures of political preference—ideology, party identity, and voting—that implicit measures are not statistically significant, or are, at most, very weak predictors. More importantly, these implicit measures are, in all cases, far less powerful predictors than explicit prejudice measures. In short, implicit measures fail to contribute additionally to the strong associations between measures of explicit sexism and, in particular, explicit racism, and political preferences and behaviors.

There are at least two limitations to our study. First, our sample was somewhat nonrepresentative of the U.S. population, having a surfeit of women, blacks, and those with higher levels of education. We therefore ran all our models with controls for the demographic characteristics. This had no effect on the substantive findings. Secondly, the intercorrelations between the two measures of implicit bias we created for gender and race, respectively—one composed of negative traits and the other composed of positive traits—were both null. Despite this, we did observe a few effects of moderate size (e.g., a black/white difference on implicit bias against blacks of about one third of a standard deviation). In addition, consistent with the prior literature (e.g., [38]), our measures of implicit racial bias were weakly correlated with measures of explicit prejudice in the expected directions.

A final, and unexpected, finding is that the significant effects of implicit measures were all for the ratings of the positive traits. This indicates that implicit responses with regard to positive traits, at least as captured by the evaluative priming paradigm, are more informative regarding bias. To the degree that implicit prejudice has an association with political preferences, it appears to result from de-emphasizing the positive rather than emphasizing the negative. Refining the implementation of implicit measures to focus on how subjects respond to positive traits may improve our understanding of how implicit biases are associated with political preferences.

## 6. Conclusions

The search for new paradigms that can accurately predict voter preferences remains a keen topic for the social sciences, particularly in the light of the somewhat unexpected outcomes in 2016 of both the U.S. election and the U.K.’s Brexit referendum. Implicit measures have been touted as a possible route by which the true feelings and/or subjective biases of voters can be uncovered, especially when sections of the electorate prefer to either not reveal their intentions to pollsters, or to conform to social norms about race and gender when asked explicitly. In this paper, we explored the utility of one of the more flexible implicit paradigms in cognitive psychology—evaluative or affective priming—to predict political attitudes and actual voting choices more effectively than explicit measures of racial or gender prejudices. Contrary to our expectation, we observed that measures of implicit bias are, in fact, less strongly associated with political attitudes and voting than explicit measures of sexist attitudes and modern racism. While this does not rule out the possibility that other implicit paradigms may have greater predictive validity, our data strongly suggest that explicit prejudice has, in today’s digital era, a greater association with political preferences than implicit stereotyping.

## Figures and Tables

**Table 1:** Descriptive statistics for our sample.

Characteristic	Percentage (%)
Female	65
Black	34
Other race	6
Age 41–60	42
Age 61+	14
Some college	42
College degree	33
Graduate degree	10
2nd income quartile	21
3rd income quartile	28
4th income quartile	21

Notes: Percentages were rounded to the nearest whole number.

**Table 2:** OLS models comparing the effects of implicit bias and explicit prejudice on conservative identity.

	Conservative Identity (z-Score)	Conservative Identity (z-Score)	Conservative Identity (z-Score)	Conservative Identity (z-Score)	Conservative Identity (z-Score)
			Panel A		
Implicit bias against females for undesirable traits (z-score)	−0.02			−0.02	
Implicit bias against females for desirable traits (z-score)		0.03			0.01
Explicit sexism (z-score)			0.35 ***	0.35 ***	0.35 ***
*R* ^2^	0.11	0.11	0.21	0.21	0.21
			Panel B		
Implicit bias against blacks for undesirable traits (z-score)	0.01			−0.02	
Implicit bias against blacks for desirable traits (z-score)		0.08 **			0.07 *
Explicit racism (z-score)			0.34 ***	0.34 ***	0.33 ***
*R* ^2^	0.11	0.11	0.19	0.19	0.20

Notes: All models control for: gender, race, age group, level of education, and income quartile. *n* = 1076. Significance levels: * 5%, ** 1%, *** 0.1%.

**Table 3:** Logit models comparing the effects of implicit bias and explicit prejudice on Republican identity.

	Republican Identity	Republican Identity	Republican Identity	Republican Identity	Republican Identity
			Panel A		
Implicit bias against females for undesirable traits (z-score)	−5.0			4.3	
Implicit bias against females for desirable traits (z-score)		4.4			1.9
Explicit sexism (z-score)			27.9 ***	27.8 ***	27.7 ***
CCC (%)	71.4	71.6	77.2	77.2	76.9
			Panel B		
Implicit bias against blacks for undesirable traits (z-score)	−1.4			−3.2	
Implicit bias against blacks for desirable traits (z-score)		6.8 *			5.3 *
Explicit racism (z-score)			31.2 ***	31.5 ***	30.7 ***
CCC (%)	70.2	71.4	77.0	77.2	77.0

Notes: Entries are average marginal effects, given in percentage points, of moving from 1sd below the mean to 1sd above the mean. All models control for: gender, race, age group, level of education, and income quartile. *n* = 986. Significance levels: * 5%, *** 0.1%.

**Table 4:** Logit models comparing the effects of implicit bias and explicit prejudice on voting for Romney.

	Voted Romney	Voted Romney	Voted Romney	Voted Romney	Voted Romney
			Panel A		
Implicit bias against females for undesirable traits (z-score)	−5.1			−5.0	
Implicit bias against females for desirable traits (z-score)		5.6 *			4.0
Explicit sexism (z-score)			23.7 ***	23.7 ***	23.3 ***
CCC (%)	72.7	73.7	77.2	77.0	77.7
			Panel B		
Implicit bias against blacks for undesirable traits (z-score)	−1.0			−2.0	
Implicit bias against blacks for desirable traits (z-score)		7.5 **			6.1 *
Explicit racism (z-score)			31.4 ***	31.5 ***	30.9 ***
CCC (%)	71.7	73.1	78.1	78.7	78.0

Notes: Entries are average marginal effects, given in percentage points, of moving from 1sd below the mean to 1sd above the mean. All models control for: gender, race, age group, level of education, and income quartile. *n* = 942. Significance levels: * 5%, ** 1%, *** 0.1%.

## Appendix A

The statement that was shown to subjects prior to the experiment:

You are invited to participate in this study titled ‘Survey on Voting Behaviour’. This study is being done for research purposes only. We intend to examine the mental process involved in the selection of a presidential candidate during elections. The survey and reaction time test will take approximately 30 min to complete. This reaction time test will consist of several trials in which you will see a word flashing in middle of the computer screen. This word will depict a personality trait in the presidential candidate and can be either desirable or an undesirable trait in a candidate.

If the trait is undesirable, press ‘I ‘key on the keyboard and if it is desirable, press the ‘E’ Key. It is important that you respond as quickly as you can and BEFORE the orange rectangle around the word disappears. If you make a mistake you will see a cross appear and you will be asked to press the correct key. If you are too slow, a warning message will appear.

Now please hold the finger of your left hand over the ‘E’ key and the finger of your right hand over ‘I’ key.

Remember, as soon as you see a word showing an undesirable trait press ‘I’ key and as soon as you see a word showing a desirable trait press the ‘E’ key. Please remember to respond as quickly as possible. Let’s begin with some practice trials.

## Appendix B

Below is the 7-item modern racism scale.

**Discrimination against blacks is no longer a problem in the United States**.
It is easy to understand the anger of black people in America.*
Blacks have more influence upon school desegregation plans than they ought to have.
Blacks are getting too demanding in their push for equal rights.
Blacks should not push themselves where they are not wanted.
Over the past few years, blacks have gotten more economically than they deserve.
Over the past few years, the government and news media have shown more respect to blacks than they deserve.

Response scale is 1, strongly disagree, to 5, strongly agree.

* Reverse scored item

**Below is the 11-item Neosexism Scale.**

Discrimination against women in the labor force is no longer a problem in the U.S.
I consider the present employment system to be unfair to women.
Women shouldn’t push themselves where they are not wanted.
Women will make more progress by being patient and not pushing too hard for change.
It is difficult to work for a female boss.
Women’s requests in terms of equality between the sexes are simply exaggerated.
Over the past few years, women have gotten more from the government than they deserve.
Universities are wrong to admit women in costly programs such as medicine, when in fact a large number will leave their jobs after a few years to raise their children.
In order not to appear sexist, many men are inclined to overcompensate women.
Due to social pressures, firms frequently have to hire underqualified women.
In a fair employment system, men and women would be considered equal. *

* Reverse scored item.

Response scale is 1, strongly disagree, to 5, strongly agree.
